# Dysregulated Adaptive Immune Responses to SARS-CoV-2 in Immunocompromised Individuals

**DOI:** 10.3390/microorganisms13051077

**Published:** 2025-05-06

**Authors:** Núria Mayola Danés, Demi Brownlie, Rebecca Folkman, Anna Nordlander, Kim Blom, Renata Varnaite, Julia Niessl, Oskar Karlsson Lindsjö, Sandra Söderholm, Mira Akber, Puran Chen, Marcus Buggert, Andreas Bråve, Jonas Klingström, Piotr Nowak, Nicole Marquardt, Klara Sondén, Ola Blennow, Sara Gredmark-Russ

**Affiliations:** 1 Center for Infectious Medicine, Department of Medicine Huddinge, Karolinska Institutet, 141 52 Huddinge, Sweden; 2 Center for Hematology and Regenerative Medicine, Department of Medicine Huddinge, Karolinska Institutet, 141 52 Huddinge, Sweden; 3 Department of Infectious Diseases, Karolinska University Hospital, 141 86 Stockholm, Sweden; 4 Department of Laboratory Medicine, Karolinska Institutet, 141 52 Huddinge, Sweden; 5 Public Health Agency of Sweden, 171 65 Solna, Sweden; 6 Department of Clinical Microbiology, Umeå University, 901 87 Umeå, Sweden; 7 Division of Molecular Medicine and Virology, Department of Biomedical and Clinical Sciences, Linköping University, 581 83 Linköping, Sweden; 8 Department of Medicine Huddinge, Karolinska Institutet, 141 52 Huddinge, Sweden; 9 Department of Medicine Solna, Karolinska Institutet, 171 77 Stockholm, Sweden; 10 Laboratory for Molecular Infection Medicine Sweden, Umeå University, 901 87 Umeå, Sweden

**Keywords:** SARS-CoV-2, COVID-19, immunocompromised patients, immunosuppression, cellular immunity, humoral immunity, T cell, B cell

## Abstract

The SARS-CoV-2 virus poses a significant risk to immunocompromised patients, who display weakened immunity and reduced seroconversion following infection and vaccination. In this study, we recruited 19 hospitalized patients with immune disorders (ImCo) and 4 immunocompetent controls (ICC) with COVID-19. We evaluated their serological, humoral, and cellular immune responses at <30 days and >90 days post-symptom onset. ICC patients showed robust B and T cell responses against SARS-CoV-2, indicated by detectable antibody levels, memory antibody-secreting cells (mASCs) towards the spike protein and spike-specific CD4^+^ and CD8^+^ T cells. ImCo patients showed impaired immune responses, with lower levels of B cell responses. Further subdivision of the ImCo patients demonstrates that solid organ transplant (SOT) patients generated B cell responses similar to ICC patients, whereas the other ImCo patients, including patients with hematological malignancies and anti-CD20 therapy, did not. Absolute T cell numbers and spike-specific CD4^+^ and CD8^+^ T cell responses were low in the ImCo patients at <30 days but increased at later time points. Our findings suggest that even when B cell responses were reduced, patients could present a T cell response, suggesting a more successful line of passive immunization for immunocompromised individuals focusing on boosting T cell responses.

## 1. Introduction

The SARS-CoV-2 pandemic has highlighted the increased risks of viral infections for immunocompromised individuals. While some reports suggest higher mortality rates in this population, others indicate that mortality and disease severity vary depending on the type of immunosuppression [1]. For instance, patients with hematologic malignancies, solid organ transplantation (SOT), rheumatic disease, and solid tumors experience higher rates of hospitalization, ICU admissions, and mortality due to COVID-19 [2]. Although mortality and hospitalization rates decreased in solid and hematologic cancer patients during the circulation of the Omicron-lineage (B.1.1.529) SARS-CoV-2 variant, unvaccinated individuals displayed mortality and morbidity similar to previous variants [3]. These trends highlight the critical role of the immune system and pre-existing immunity to SARS-CoV-2 in determining outcomes, particularly in immunocompromised populations. Thus, further exploration into the immune response to SARS-CoV-2 infection in these groups is important.

SARS-CoV-2-specific antibody titers in immunocompetent patients are normally proportional to the severity of disease [4] and following the peak at day 15–28 post-infection, the antibody titers gradually decreased over time and stabilized after 6 months [5]. Robust SARS-CoV-2-specific T cell responses have also been observed 8–12 months post-infection [6,7,8]. Although SARS-CoV-2-specific mRNA vaccines have greatly alleviated mortality and disease severity in immunocompetent patients [9], immunocompromised patients exhibit lower seroconversion rates [10]. Studies on SOT patients show generation of neutralizing antibodies after two doses of the same mRNA vaccine [11], but their overall antibody response remains decreased compared to immunocompetent patients [12,13,14].

Patients on B-cell-depleting medication/anti-CD20 treatment have reduced spike-specific antibodies after SARS-CoV-2 mRNA vaccination, even after a third dose [15,16]. Some patients on anti-CD20 treatment are able to mount a response if the immunosuppression is reduced and the B cells have recovered [17]. Even in the absence of B cells, patients can mount functional T cell responses. T cell responses post-vaccination have been observed in those with X-linked agammaglobulinemia and in patients on anti-CD20 treatment [18]. Patients with hematological malignancies may also develop robust T cell responses after vaccination, with functional CD8^+^ T cell responses correlating with outcomes and survival from COVID-19 [19,20].

The continued emergence of new SARS-CoV-2 variants places immunocompromised individuals at ongoing risk, despite vaccination and previous infection, making them vulnerable to severe disease and persisting infection [21]. In the current study, we aimed to characterize the cellular and humoral immune profile in immunocompromised patients including solid organ transplant (SOT) patients on calcineurin inhibitor tacrolimus, hematological malignancies, patients receiving B-cell-depleting medication (anti-CD20) and TNF-α inhibitors. We evaluated the immune responses at <30 days and >90 days after symptom onset in order to understand the earlier and convalescent immune responses in immunocompromised patients. We assessed antibody levels to the spike and nucleocapsid protein, virus neutralization, together with FluoroSpot assays to measure memory antibody-secreting cells (mASCs) towards the spike protein and activation-induced marker (AIM) assays with peptides covering the full spike sequence, to understand the complex immune response in these patient groups.

## 2. Materials and Methods

### 2.1. Study Subjects and Sampling

Twenty-three patients hospitalized due to PCR-confirmed SARS-CoV-2 infection, nineteen with an immunosuppressive condition and four immunocompetent controls, were included at Karolinska University Hospital, Stockholm, Sweden between February and December 2021. During the first part of the study period, Alpha variant (B.1.1.7) of SARS-CoV-2 was dominating in Sweden. In June 2021, a shift towards Delta variants (B.1.617.2) occurred and by July 2021, nearly 100% of sequenced viruses were of this variant [22]. Samples collected were divided into the following time points <30 days (median: 16 days, range 4–27 days) and >90 days (median: 176 days, range 99–265 days). Patients were assessed for severity using the World Health Organization (WHO) ordinal clinical scale. The WHO severity scale ranges from 0–10 with 0 meaning no evidence of infection and 10 being death [23]. This study was approved by the Swedish Ethical Review Authority, approval number 2020-01558 and 2021-00603. All patients provided informed written consent. Venous blood was collected in lithium-heparin tubes, EDTA tubes, and serum clot activators (Vacuette, Monroe, NC, USA). PBMCs were isolated using density gradient centrifugation using SepMate tubes (STEMCELL technologies, Vancouver, BC, Canada) and were either used fresh or cryopreserved in fetal bovine serum (FBS) supplemented with 10% dimethylsulfoxide (DMSO). Serum tubes were allowed to stand upright for 2 h at room temperature (RT) before serum was isolated by centrifugation at 2000× *g* for 10 min and stored at −80 °C for later analysis.

### 2.2. Activation-Induced Marker (AIM) Assay

PBMCs were thawed in RPMI 1640 medium supplemented with 10% FBS, 1% L-glutamine and 1% penicillin/streptomycin (R10, complete medium). Cells were resuspended in R10 and rested for 3 h at 37 °C at a concentration of 2 × 10^6^ cell/well in a 96-well U-bottom plate. CXCR5-BB515 (BD Biosciences, New Jersey, NJ, USA) and anti-CD40 (unconjugated, Miltenyi Biotec, Bergisch Gladbach, Germany) were added to each well and incubated for 15 min. Cells were then stimulated with 500 ng/mL S-Comp peptide (Miltenyi Biotec), while unstimulated negative control cells received an equivalent concentration of DMSO. Following an 18 h incubation, cells were stained as described under *Flow Cytometry*.

The response was quantified by dividing the percentage of spike-specific cells in the peptide-stimulated sample by the percentage of spike-specific cells from the unstimulated control sample. If the peptide-stimulated response was more than double that of the unstimulated control response, the donor was classified as a ‘responder’; otherwise, they were considered a ‘non-responder’. For further analysis of T cell helper and memory cell subsets, at least 10 cells were required in the maternal gate.

### 2.3. Flow Cytometry

For TruCount analysis, 50 μL of whole blood from EDTA tube was added to a TruCount absolute counting tube (BD Biosciences) along with the following antibody cocktail: BD Multitest 6-color TBNK Reagent with BD TruCount tubes (BD Biosciences), HLADR-BV786 (BioLegend, San Diego, CA, USA), CD15-Pacific Blue (BioLegend), CD193-BV605 (BioLegend), CD123-BUV395 (BD Biosciences), CD14-PE-Cy5 (ThermoFisher Scientific, Waltham, MA, USA) and incubated for 20 min at RT. Red blood cells were lysed (1X BD FACS lysing solution) following the manufacturer’s instructions. Data were acquired on a BD FACSymphony A5 (BD Biosciences). The gating strategy is shown in Figure S1.

For AIM assays, cells were stimulated with peptides for 18 h and then were washed twice with FACS buffer (PBS, 2% FCS, 2mM EDTA) and transferred to a 96-well V-bottom plate. The cells were stained with Aqua LIVE/DEAD fixable dead cell stain (ThermoFisher) for 10 min at RT. Next, antibodies against CCR4-BB700 (BD Biosciences), CCR7-APC-Cy7 (BioLegend), CCR6- BUV737 (BD Biosciences), CXCR3 (BD Biosciences) were added and incubated for a further 10 min at 37 °C in FACS wash. CD40L-BV421 (Miltenyi Biotec), 4-1BB-PE-Cy7 (BioLegend), CD4-BUV496 (BD Biosciences), CD14-BV510 (BioLegend), CD19 (BioLegend), CD45RA-BV570 (BioLegend), CD69-BUV563 (BD Biosciences), CD3 (BD Biosciences), and CD8-BUV395 (BD Biosciences) were added in Brilliant Stain Buffer Plus (BD Biosciences) and incubated for 30 min at RT in the dark. The antibodies selected for AIM assay provided an understanding of the memory and helper subsets as well as the activation markers for both CD4^+^ and CD8^+^ T cells. Cells were then washed with FACS buffer, fixed in 1% paraformaldehyde (Biotum) in PBS for 20 min at RT, washed again, and resuspended in FACS buffer. Data acquisition was performed using a BD FACSymphony A3 (BD Biosciences), and the data were analyzed with FlowJo software (BD Biosciences) version 10.9.0.

### 2.4. Spike and ACE2 Inhibition Assay

Pseudo-neutralizing titers (antibodies capable of blocking spike-ACE2 binding) against SARS-CoV-2 were quantified by V-PLEX SARS-CoV-2 (Panel 2(ACE2) Meso Scale Diagnostics, Rockville, MD, USA) according to the manufacturer’s instructions. Results are expressed in μg/mL.

### 2.5. Meso Scale

Binding IgG against SARS-CoV-2 spike, RBD and N were quantified by V-PLEX SARS-CoV-2 (Panel 2, Meso Scale Diagnostics, Rockville, MD, USA) according to the manufacturer’s instructions. Results are reported in binding arbitrary units (BAU)/mL.

### 2.6. Polymerase Chain Reaction (PCR)

SARS-CoV-2 RT-PCR targeting the RNA dependent RNA polymerase (RdRp)-genes were used to detect the presence of SARS-CoV-2 RNA in patient samples, the primers are modified from previously described [24]. The sensitivity of the test is as described in [24], 3.2 RNA copies/reaction. The PCR reaction of 25 μL contained 5 μL of RNA, 6.25 μL of 4 × reaction buffer TaqMan FAST Virus 1-step MM (Applied biosystems, Thermo Fisher), 4 μL primers and probe mix and water (shown in Table S1). Thermal cycling was performed at 55 °C for 5 min for reverse transcription, followed by 95 °C for 20 sec and then 45 cycles of 95 °C for 3 s and 60 °C for 30 s in a StepOnePlus™ instrument from Applied Biosystems (Waltham, MA, USA). The threshold for a negative result was set at a CT-value of over 40 cycles out of 45.

### 2.7. Viral Cultures for Cytopathic Effect (CPE) Assessment

Patient samples were incubated with *Vero E* cells in 24 well plates for 4 days. The plates were then read with a microscope for CPE. Samples with CPE were inactivated with TRIzol and analyzed with PCR. The threshold for a negative result was set at a CT-value above 40. Samples with lower CT-values after isolation were assessed as positive. Samples without CPE after 10 days were analyzed with PCR and assessed as negative if the CT-value was the same or higher than the starting sample.

### 2.8. Memory B Cell FluoroSpot Assay

The B cell FluoroSpot kit (Mabtech, Nacka Strand, Sweden) was used to detect spike-specific memory B cell-derived mASCs, with total IgG measured as control to assess the presence of functional mASCs. Cryopreserved PMBCs were thawed, resuspended in complete R10 medium and counted using the Countess 3 (ThermoFisher). Polyclonal B cell stimulation was conducted for 3 days with 1 µg mL^−1^ R848 and 10 ng mL^−1^ rIL-2 (Mabtech B cell StimPack) in R10 medium at 37 °C in 5% CO_2_. This stimulation allowed memory B cells (mBCs) to differentiate into mASCs.

Low autofluorescent polyvinylidene difluoride membrane plates, previously activated for 30 s to one minute with 35% ethanol, were washed thoroughly with water and coated overnight with capture anti-human IgG mAb (MT145) (Mabtech). After washing with PBS, the plates were blocked for 30 min with R10 medium. Stimulated mASCs were added to the plates and incubated for 24 h at 37 °C and 5.5% CO_2_. Following incubation, the plates were washed with PBS, and spots were stained according to FluoroSpot Path: SARS-CoV-2 (Spike) Human IgG instructions (Mabtech). Spots were detected and counted with an IRIS FluoroSpot reader and Mabtech Apex software version 1.1 build 59 (Mabtech). The threshold of positivity was defined as double the median of spike-specific spots of PBMCs of pre-pandemic healthy donors collected with approval of the Regional Ethical Review Board in Stockholm (2016/1415-32).

### 2.9. Statistical Analysis

Statistical analysis was performed using GraphPad Prism 10.4.1 (GraphPad Software, San Diego, CA, USA). Data sets were analyzed using the Wilcoxon signed-rank test or the Mann–Whitney *U*-test.

Statistical significance was considered where *p*-values < 0.05 (* *p* < 0.05; ** *p* < 0.01; *** *p* < 0.001).

## 3. Results

### 3.1. Patient Characteristics and Outcome

We assessed the immune response from immunocompromised patients (ImCo) with COVID-19 as summarized in Table 1. A total of 7 of the patients had undergone SOT (*n* = 7), and 12 of the patients had either hematological disease (*n* = 5), anti-CD20 medication (*n* = 6), or TNF-α inhibitor treatment (*n* = 1). We also included immunocompetent controls (ICC) (*n* = 4). Patient characteristics, vaccination and treatments are shown in Table 1. Samples were collected at <30 days after symptom debut (median = 16 days, range = 4–27) and at >90 days after symptom debut (median = 176, range = 99–265).

A total of 10 patients received 1 to 2 doses of the SARS-CoV-2 vaccine before inclusion in this study (Table 1).

Treatment for COVID-19 included monoclonal antibodies (mAbs) targeting the spike protein (bamlanivimab and casirivimab/imdevimab) (median days after symptom debut: 7 days, range 6–19), convalescent plasma from recovered patients (median days after symptom debut: 15.5 days, range 5–86), corticosteroids (median days after symptom debut: 8 days, range 4–37), and anti-viral remdesivir (median days after symptom debut: 6 days, range 3–70). Treatments are outlined in Figure S2A.

The median time of hospitalization was similar for the ICC and the ImCo patients (Table 2). In total, four patients were admitted to the ICU, two of the ImCo and two of ICC patients (Table 2). All ImCo patients except three, and three out of four ICC patients were either hospitalized with moderate disease with either no oxygen therapy or oxygen therapy by mask, levels 4 and 5 according to the WHO clinical severity scale [23]. Three ImCo patients were classified as mild disease with assistance needed (level 3) (Table 2). And one ICC patient was hospitalized with oxygen with non-invasive ventilation on high flow (level 6) (Table 2) [23]. When assessing airway (sputum or nasopharyngeal) samples with in vitro culture of replicating virus, 9 patients had virus positive cultures at the first sampling time, determined by the detection of SARS-CoV-2 RNA in the cultures (*n* = 10) (Figure S2C), all but 1 had a CT-value on or below 25 in the original airway sample. Two of the cultures from the positive airway samples had visual cytopathic effect (CPE), from which both of the original samples had CT-values below 22 (Figure S2C). In samples with detectable SARS-CoV-RNA, but no CPE, the CT-values were slightly higher (on or above 23) in the original airway samples (Figure S2C).

### 3.2. Immune Cell Composition in Immunosuppressed Patients During COVID-19

**Figure 1:**
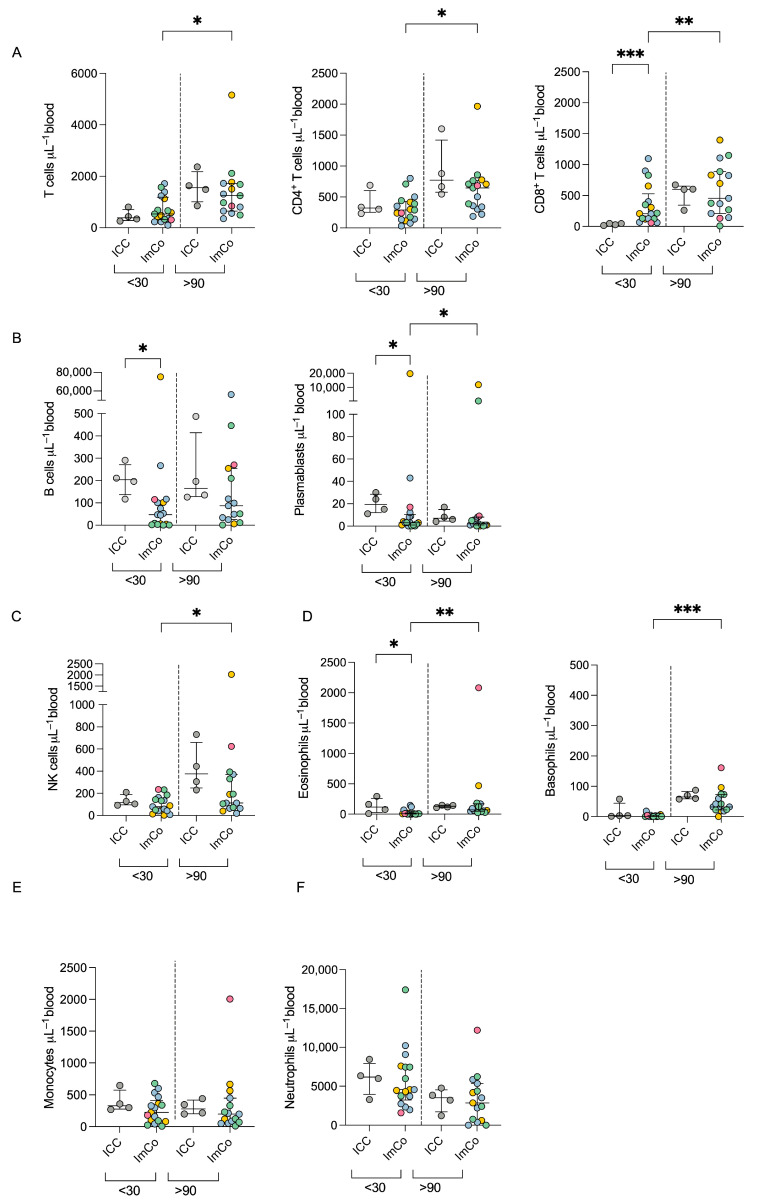
Absolute cell number count in COVID-19 patients. (**A**) Total numbers of total T (CD3^+^ CD19^−^), CD4^+^, and CD8^+^ T cell numbers per μL of whole blood at <30 days (ICC *n* = 4, ImCO *n* = 17) and >90 days (ICC *n* = 4, ImCO *n* = 15). Plots depict median with IQR. (**B**) Total B cell (CD3^−^ CD19^+^) and plasmablast numbers per μL of whole blood at <30 days (ICC *n* = 4, ImCO *n* = 17) and >90 days (ICC *n* = 4, ImCO *n* =15). Plots depict median with IQR. (**C**) NK cell (CD16^+^ CD56^+^) numbers per μL of whole blood at <30 days (ICC *n* = 4, ImCO *n* = 17) and >90 days (ICC *n* = 4, ImCO *n* = 15). Plots depict median with IQR. (**D**) Eosinophil (CD45^+^CD15^+^CD193^+^ CD14^−^) and basophil (CD45^+^SSC^low^Lineage^−^(CD3^−^CD19^−^CD14^−^CD4^−^CD16^−^CD56^−^) CD123^+^) numbers per μL of whole blood <30 days (eosinophils: ICC *n* = 4, ImCO *n* = 17) (basophils: ICC *n* = 4, ImCO *n* = 17) and >90 days (eosinophils: ICC *n* = 4, ImCO *n* = 15) (basophils: ICC *n* = 4, ImCO *n* = 15). Plots depict median with IQR. (**E**) Monocyte number (CD14^+^ CD56^+^) per μL of whole blood at <30 days (ICC *n* = 4, ImCO *n* = 17) and >90 days (ICC *n* = 4, ImCO *n* = 15). Plots depict median with IQR. (**F**) Neutrophil numbers per μL of whole blood at <30 days (ICC *n* = 4, ImCO *n* = 17) and >90 days (ICC *n* = 4, ImCO *n* = 15). Gray circles represent ICC patients, blue circles represent SOT patients, yellow circles represent immunosuppression due to malignancy, green circles represent patients on anti-CD20 therapy, and pink circles represent patients on TNF-α inhibitors. Plots depict median with IQR. Statistical significance within paired groups was assessed with the Wilcoxon signed-rank test * *p* < 0.05; ** *p* < 0.01; *** *p* < 0.001. Statistical significance amongst different groups was assessed with the Mann–Whitney U test * *p* < 0.05; ** *p* < 0.01; *** *p* < 0.001.

We first tracked the major lymphoid and myeloid cell subsets in the peripheral blood in immunocompromised donors and healthy controls, using TruCount absolute counting tubes. We revealed variations in cell numbers at the early (<30 days) and late (>90 days) time points of the infection with SARS-CoV-2 (Figure 1A–F; gating strategy can be found in Figure S1). Generally, the absolute numbers of T cells, and NK cells were lower at the <30 days time point after infection but increased at later time points in the ImCo patients (Figure 1A,C,D). The absolute number of CD8^+^ T cells was higher in the ImCo that the ICC patients at the <30 day time point (Figure 1C).

The absolute B cell and plasmablast counts were higher in the ICC patients as compared to the ImCo at the early time point, and the levels of B cells were stationary over time in both patient groups (Figure 1B). The majority of ImCo patients had low levels of plasmablasts at both <30 and >90 days, except one patient with chronic lymphocytic leukemia and one patient on anti-CD20 medication (Figure 1B). Eosinophil and basophil levels present a slight increase in numbers at the >90 day time point (Figure 1D) Monocyte and neutrophil levels were stable at early and late time points for both ICC and ImCo (Figure 1E,F). Classical monocytes represented most of the monocytes in both ICC and ImCo (Figure S1B). In both groups, an increase in both plasmacytoid dendritic cells and classical dendritic cells was observed at the later time point (Figure S1C). The B cell, CD4^+^, and CD8^+^ T cell numbers are consistent with previous reports in immunocompetent patients with acute COVID-19 [25].

As expected, ICC patients present higher numbers of B cells and overall number of immune cells in comparison to the ImCo. Due to said expected differences in immune cell composition among ImCo patients and ICC, we proceeded with further exploration of the functional capabilities of the cells in the respective patient group.

### 3.3. SARS-CoV-2-Specific IgG Levels Differ Across Immunocompromised Patient Groups over Time

**Figure 2:**
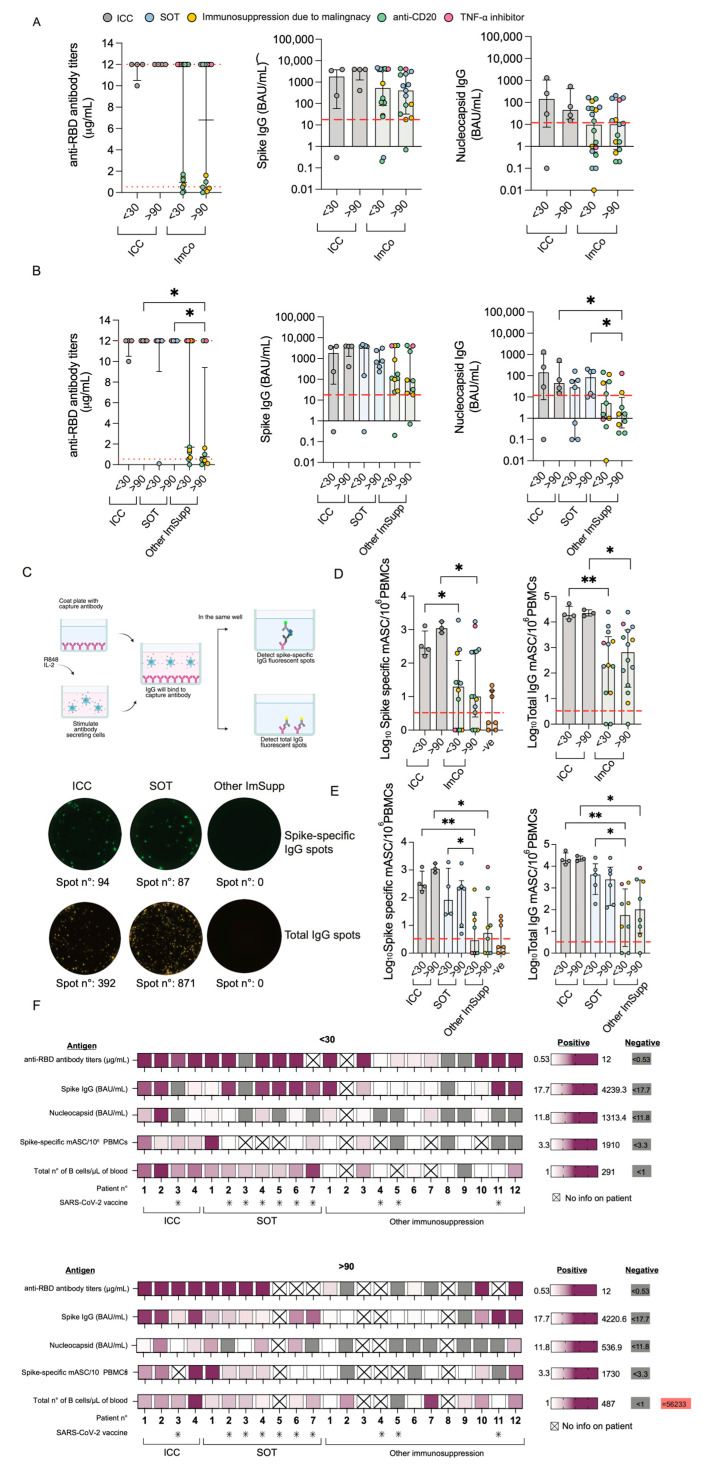
Antibody and B cell responses during COVID-19. (**A**) Spike/ACE2-blocking antibodies at <30 days (ICC (*n* = 4) vs. ImCo (*n* = 18)) and >90 days (ICC (*n* = 4) vs. ImCo (*n* = 15)). The dotted lines denote the positive threshold (≥0.533 μg/mL) and upper detection limit (12 μg/mL). Spike IgG levels at <30 in ICC (*n* = 4) and ImCo (*n* = 18) and >90 days in ICC (*n* = 4) and ImCo (*n* = 15). The dotted line denotes the positive threshold (17.7 BAU/mL). Nucleocapsid-specific IgG at <30 days in ICC (*n* = 4) and ImCo (*n* = 18) and >90 days in ICC (*n* = 4) and ImCo (*n* = 15). The dotted line denotes the positive threshold (11.8 BAU/mL). (**B**) Spike/ACE2-blocking antibodies at <30 days in ICC (*n* = 4), SOT (*n* = 7), and Other ImSupp (*n* = 11) and >90 days in ICC (*n* = 4), SOT (*n* = 6), and Other ImSupp (*n* = 9). The dotted lines denote the positive threshold (≥0.533 μg/mL) and upper detection limit (12 μg/mL). Spike IgG levels at <30 days in ICC (*n* = 4), SOT (*n* = 7), and Other ImSupp (*n* = 11) and >90 days in ICC (*n* = 4), SOT (*n* = 6), and Other ImSupp (*n* = 9). The dotted line denotes the positive threshold (17.7 BAU/mL). Nucleocapsid-specific IgG at <30 days in ICC (*n* = 4), SOT (*n* = 7), and Other ImSupp (*n* = 11) and >90 days in ICC (*n* = 4), SOT (*n* = 6), and Other ImSupp (*n* = 9). The dotted line denotes the positive threshold (11.8 BAU/mL). (**C**) Schematic overview of the FluoroSpot assay for detection memory antibody-secreting cells and representative FluoroSpot images with Spike-specific IgG spots (upper panel) and total IgG spots (lower panel) in each individual patient group. Created in BioRender Mayola, N. (2025) https://BioRender.com/p40r113 (accessed on 1 March 2025). (**D**) Spike-specific mASCs per 1 million plated PBMCs and total IgG-secreting mASCs per 1 million plated PBMCs at <30 days and >90 days in ICC and ImCo as well as the pre-pandemic PBMCs of healthy subjects. Red line depicts threshold of positivity, defined by the double the median of spike-specific background spots of the pre-pandemic samples. Patients plotted with 0.01 spots/1M PBMCs, had 0 spots. The plots present log10 transformed data in a linear Y axis and depicting median with IQR. (**E**) Spike-specific mASCs per 1 million plated PBMCs and total IgG-secreting mASCs per 1 million plated PBMCs at <30 days and >90 days in ICC, SOT, and other immunosuppression as well as the pre-pandemic PBMCs of healthy subjects. Red line depicts threshold of positivity, defined by the double the median of spike-specific background spots of the pre-pandemic samples. Patients plotted with 0.01 spots/1M PBMCs, had 0 spots. The plots present log10 transformed data in a linear Y axis and depicting median with IQR (**F**) Heatmaps summarizing the Spike/ACE2-blocking antibodies, IgG against spike, S1 RBD and nucleocapsid, spike-specific mASCs, total number of B cells/μL of whole blood data, and at least one dose of vaccine before inclusion in this study in individual patients at <30 and >90 after symptom debut. Patients who received at least one dose of the vaccine are marked with a star. Statistical significance within paired groups was assessed with the Wilcoxon signed-rank test * *p* < 0.05; ** *p* < 0.01. Statistical significance amongst different groups was assessed with the Mann–Whitney U test * *p*< 0.05; ** *p*< 0.01.

First, we set off to assess the function of B cells and plamablasts. We examined the levels and dynamics of SARS-CoV-2-specific Spike/ACE2-blocking antibodies and IgG at the early (<30 days) and late (>90 days) time points of the infection. All ICC patients had Spike/ACE2-blocking antibodies towards SARS-CoV-2 at both <30 and >90 days (Figure 2A). In the ImCo patient group, 10/17 patients had Spike/ACE2-blocking antibodies at <30 days after infection, and 6/12 patients had Spike/ACE2-blocking antibodies at >90 days (Figure 2A). A total of 7 of the ImCo and 1 of the ICC patients had received at least 1 dose of vaccination a median of 25.5 days (range 15–116) before the <30 day sampling (Figure 2F). A total of 7 out of the 23 patients received mAbs a median of 5.5 days (range: 0–8 days) before the <30 day sampling and a median of 162 days (range: 89–177 days) before the >90 day sampling. A total of 9 of the 23 patients were administered convalescent plasma at a median of 3.5 days (range: 0–10 days) before the <30 day sampling and at a median of 180 days (range: 126–229) before the >90 day sampling (Figure S2). The distribution of mAbs and convalescent plasma likely contributed to the Spike/ACE2-blocking antibodies at the early time point, but not at the later time point [26,27].

We also found a similar pattern of IgG levels specific to the SARS-CoV-2 spike protein (Spike), with detectable levels of IgG at >90 days in all ICC patients, and in the majority of the ImCo patients at the same time point (>90 days) (Figure 2A). As some of the patients had received treatment with mAbs, we also assessed IgG specific to the nucleocapsid (N) protein to account for the patients’ intrinsic ability to mount an antibody response to SARS-CoV-2. All but one ICC patient had seroconverted at <30 days, and all ICC patients had detectable levels of N-IgG at >90 days (Figure 2A). Fewer ImCo patients had detectable N-IgG antibodies at the <30 day time point (9/18 patients with available samples), and at the >90 days (6/17 patients with available samples) (Figure 2A), these data suggest an impaired ability of the ImCo patients to produce and maintain an adequate antibody response.

We noticed a pattern within the ImCo patients both on number of days of hospitalization and the Spike/ACE2-blocking antibodies at the later time point suggesting that there would be differences within the group. We thus further subdivided the ImCo patients into two groups partly based on immunosuppression: Solid organ transplantation (SOT) and other immunosuppression (Other ImSupp) including the patients on anti-CD20 treatment, hematological malignities and TNF-α inhibitory treatment. Interestingly, the group of Other ImSupp patients present with significantly lower the Spike/ACE2-blocking antibodies and levels of N-IgG at >90 days, whereas the levels of Spike IgG were not significantly different to ICC and SOT patients (Figure 2B).

To consider possible confounding factors, antibody levels were analyzed based on patients’ treatment with mAbs or without mAb treatment (Figure S3A–C), and treatment with convalescent plasma or without convalescent plasma treatment (Figure S3D–F). In the group of patients that did not receive mAb, 6/9 had Spike/ACE2-blocking antibodies above threshold, whereas all but one patient that received mAb treatment had detectable Spike/ACE2-blocking antibodies at <30 days after infection. At >90 days, patients that did not receive mAbs 4/7 had Spike/ACE2-blocking antibodies above threshold, whereas all but one patient with mAb treatment had detectable Spike/ACE2-blocking antibodies. Patients that received mAbs had higher levels of spike IgG, but not N-IgG levels (Figure S3A–C). In the group of patients that did not receive convalescent plasma, all 8/10 patients had Spike/ACE2-blocking antibodies above threshold. In the group that received convalescent plasma, 5/6 patients had detectable Spike/ACE2-blocking antibodies at <30 days after infection. At >90 days, only 1 of 7 patients that did not receive convalescent plasma had Spike/ACE2-blocking antibodies above threshold. Conversely, in the group that did receive convalescent plasma, 3/6 patients presented with Spike/ACE2-blocking antibodies above threshold. Patients that received convalescent plasma treatment had lower levels of Spike IgG than patients that did not receive it (Figure S3D–F). In addition, patients that received convalescent plasma treatment had lower levels of N-IgG at >90 days than ICC (Figure S3D–F).

### 3.4. Spike-Specific Memory Antibody-Secreting Cells Are Diminished in Immunocompromised Patients

To further understand the function of the cells in these patients, and to avoid confounding effects of mAb and convalescent plasma when measuring antibody levels, we next investigated the memory B cell spike-specific response. By utilizing a FluoroSpot we could detect the total IgG-secreting cells and spike-specific memory B cells after polyclonal stimulation at both <30 and >90 days (Figure 2C).

Spike-specific mASCs were consistently detected in all ICC patients at both time points, with an overall increase in numbers at >90 days (median 288, range 162–1290 mASCs/10^6^ at <30 days; median 1090, range 802–1725 mASCs/10^6^ PBMCs at >90 days) (Figure 2D). In ImCo patients, spike-specific mASC levels were over the threshold of positivity in 8 of the 12 patients at <30 days and in 11/14 at >90 days (median at <30 days: 20 mASCs/10^6^ and range: 0–1910 mASCs, median at >90 days: 10 mASCs/10^6^ and range: 0–1730 mASCs) (Figure 2D), limited by the availability of PBMCs, we were not able to perform Fluorospot for all patients. We then performed a subanalysis dividing the ImCo patients into two groups: SOT and Other ImSupp patients (Figure 2E). Consistent with our findings in the serological response, when performing a subanalysis dividing the ImCo patients into SOT and Other ImSupp, we found significantly lower levels of spike-specific mASCs at <30 days in the group of Other ImSupp patients as compared to ICC and SOT (Figure 2E), and at later time point we found lower numbers of spike-specific mASCs in the group of Other ImSupp patients as compared to ICC (Figure 2E). All serology and vaccination data in individual patients are summarized in Figure 2F.

Taken together, ImCo patients had a diverse B cell response depending on the type of immunosuppression, the SOT patients were able to mount and maintain a B cell response, whereas the Other ImSupp group, although diverse, had lower levels of Spike/ACE2-blocking antibodies N-IgG levels and spike-specific mASCs.

### 3.5. Immunocompromised Patients Display Robust Yet Variable CD4^+^ T Cell Responses

**Figure 3:**
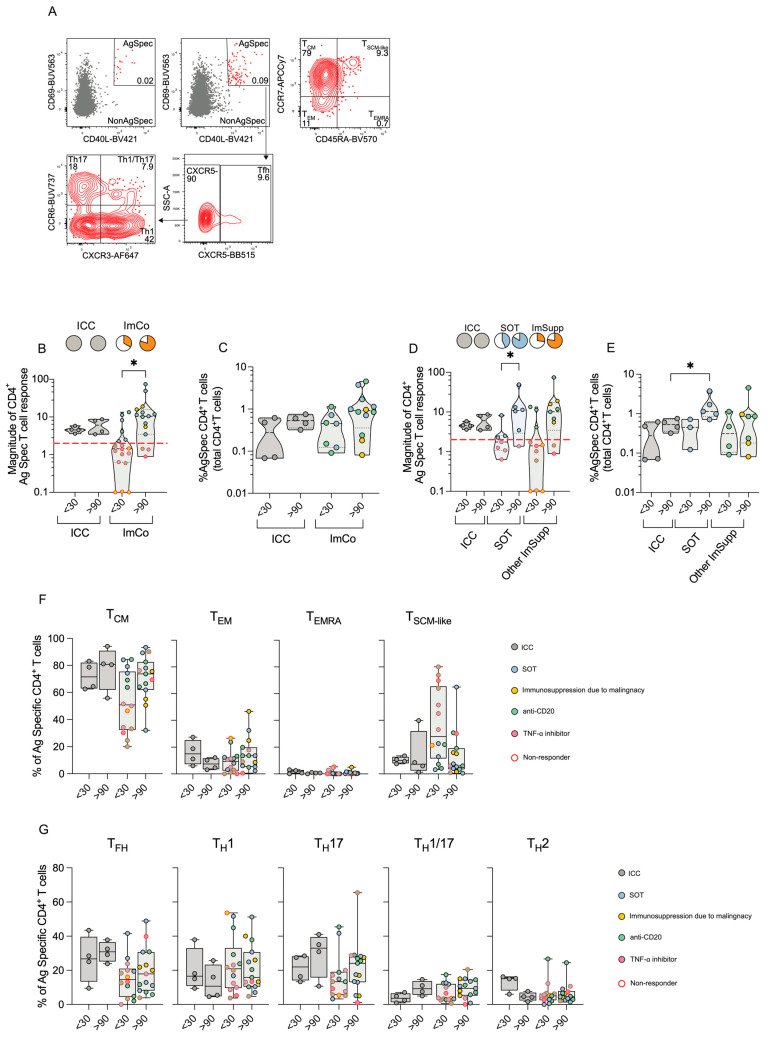
CD4^+^ T cell responses ICC and ImCo COVID-19 patients. (**A**) Representative plots and gating strategy of activation-induced markers CD69^+^ and CD40L^+^ in the CD4^+^ T cell population (unstimulated control and stimulated conditions). CD69^+^ CD40L^+^ double-positive (antigen specific cells are marked in red) cells were further divided into CD4^+^ T cell memory subsets based on expression of CXCR5, CXCR3, CCR6 and helper subsets based on expression of CCR7 and CD45RA (**B**) Magnitude of antigen-specific CD4^+^ T cells at <30 (ICC *n* = 4, ImCO *n* = 18) and >90 days (ICC *n* = 4, ImCO *n* = 15) (calculated as fold change of % of antigen-specific cells over % background of spike-specific cells in the unstimulated control). Red dotted line shows the response threshold. Pie charts show the proportion of ‘responders’ (white = ‘non-responders’). (**C**) Percentage of antigen-specific CD4^+^ T cells of total CD4^+^ T cells of ‘responders’ in ICC and ImCo at <30 (ICC *n* = 4, ImCO *n* = 7) and >90 days (ICC *n* = 4, ImCO *n* = 12). (**D**) Magnitude of antigen-specific CD4^+^ T cells at <30 (ICC *n* = 4, SOT *n* = 7, Other ImSupp *n* = 11) and >90 days (ICC *n* = 4, SOT *n* = 6, Other ImSupp *n* = 9) in ICC, SOT and Other ImSupp (calculated as fold change of % of antigen-specific cells over % background of spike-specific cells in the unstimulated control). Red dotted line shows the response threshold. Pie charts show the proportion of ‘responders’ (white = not-responding). (**E**) Percentage of antigen-specific CD4^+^ T cells of total CD4^+^ T cells of ‘responders’ in ICC, SOT and Other ImSupp at <30 (ICC *n* = 4, SOT *n* = 3, Other ImSupp *n* = 4) and >90 days (ICC *n* = 4, SOT *n* = 5, Other ImSupp *n* = 7) (**F**) Percentage of memory-specific subsets of antigen-specific CD4^+^ T cells in ICC and ImCo at <30 (ICC *n*= 4, ImCO *n* = 14) and >90 days (ICC *n* = 4, ImCO *n* = 15). (**G**) Percentage of T helper subsets of total antigen-specific CD4^+^ T cells in ICC and ImCo at <30 (ICC *n* = 4, ImCO *n* = 14) and >90 days (ICC *n* = 4, ImCO *n* = 15). Statistical significance within paired groups was assessed with the Wilcoxon signed-rank test * *p* < 0.05. Statistical significance amongst different was assessed with the Mann–Whitney U test * *p* < 0.05.

Given the variable mASC and antibody responses in the ImCo patients, we next determined their capacity to generate a T cell response to SARS-CoV-2. PBMCs from patients at early and late time points were stimulated with peptide pools covering the full spike protein sequence. Activation-induced markers (AIM) assay was used to identify and quantify spike-specific CD4^+^ T cells (CD69^+^ and CD40L^+^) via flow cytometry (gating strategy and subsequent analysis of CD4^+^ T cell memory and helper subsets are depicted in Figure 3A).

Our data revealed that ICC patients consistently mounted a robust CD4^+^ T cell response both at the <30 days and the >90 days time point (Figure 3B). Conversely, despite a only partial response in the ImCo patients at <30 days (6/18), the majority of the patients mounted a response at >90 days (12/15) (Figure 3B). Among ‘responders’, the magnitude of the CD4^+^ T cell response was comparable in the ICC and ImCO patient groups, with no significant differences in the frequencies of antigen-specific CD4^+^ T cells at <30 days (Figure 3C). During convalescence, both ICC and ImCo patients maintained similar percentages of antigen-specific CD4^+^ T cells at both time points (Figure 3C). In analogy with the previous subdivision of patients, we separated the ImCo patients into SOT and Other ImSupp. SOT patients present a clear increase in magnitude of response between the early and late time points (Figure 3D). In addition, the SOT patients also had high levels of antigen-specific CD4^+^ T cells at >90 days (Figure 3E).

For samples with sufficient numbers of antigen-specific cells, we further dissected antigen-specific CD4^+^ T cells (CD69^+^CD40L^+^, Figure 3A) by evaluating CD4^+^ T memory (Figure 3F) and helper (Figure 3G) cell subsets at both <30 and >90 days. Central memory T (T_CM_) cells were the predominant subset in both ICC and ImCo groups and both time points, followed by T_SCM-like_, T_EM_, and T_EMRA_ cells (Figure 3D,E). At <30 days, ImCo patients exhibited slightly lower T_CM_ proportions and higher T_SCM-like_ CD4^+^ cell proportions, while T_EMRA_ cell proportions remained low across all groups and time points (Figure 3D,E).

When analyzing T helper subsets, we noticed a trend of lower levels of T_FH_ cells and T_H_17 cells in the ImCo patients (Figure 3G). At the same time, there was a trend of higher levels of T_H_1 cells in this patient group (Figure 3G).

Altogether, the magnitude of the CD4^+^ T spike-specific response was lower at earlier time in the ImCo patients, with the majority of the patients not responding at the early time point. However, over time the number of ‘responders’ and the magnitude of response increase in these patients, suggesting the development of a memory response also in immunosuppressed patients.

### 3.6. CD8^+^ T Cell Responses Reveal Compensatory Immunity in Immunocompromised Patients

**Figure 4:**
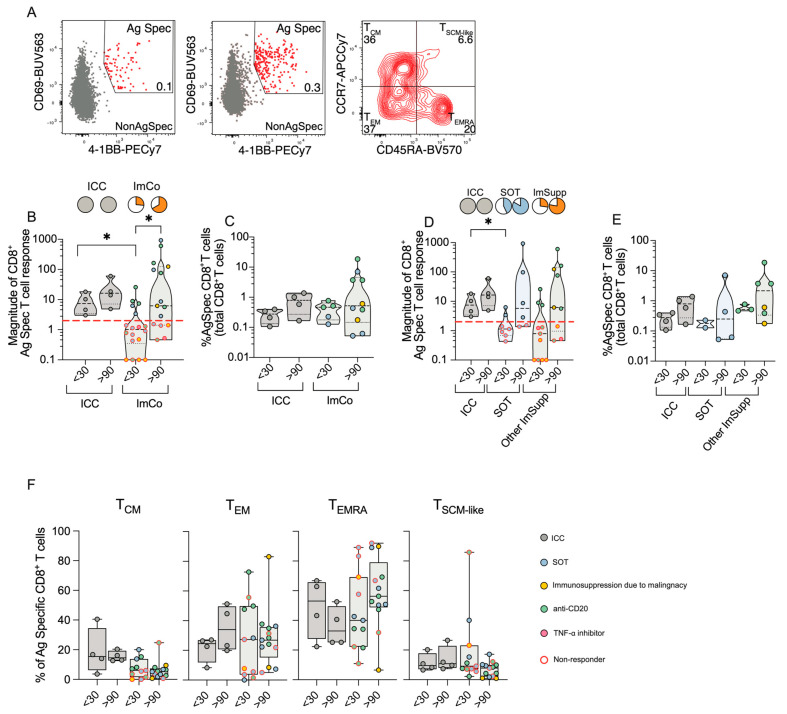
CD8^+^ T cell responses in ICC and ImCo COVID-19 patients. (**A**) Representative plots and gating strategy of activation-induced markers CD69^+^ and 4-1BB^+^ in the CD8^+^ T cell population with a unstimulated control and a spike protein stimulated condition. CD69^+^ 4-1BB^+^ double-positive (antigen-specific cells are marked in red) cells were further divided into CD8^+^ T cell memory subsets based on expression of CCR7 and CD45RA. (**B**) Magnitude of antigen-specific CD8^+^ T cells of different patient groups at <30 (ICC *n* = 4, ImCO *n* = 18) and >90 days (ICC *n* = 4, ImCO *n* = 15) in ICC and ImCo (calculated as fold change of % of antigen-specific cells over % background of spike-specific cells in the unstimulated control). Red dotted line shows the response threshold. Pie charts show the proportion of ‘responders’ (white = ‘non-responders’). (**C**) Percentage of antigen-specific CD8^+^ T cell of the total CD8^+^ T cell population of ‘responders’ in ICC and ImCo at <30 (ICC *n* = 4, ImCO *n* = 5) and >90 days (ICC *n* = 4, ImCO *n* = 10) (**D**) Magnitude of antigen-specific CD8^+^ T cells of different patient groups at <30 (ICC *n* = 4, SOT *n* = 7, Other ImSupp *n* = 11) and >90 days (ICC *n* = 4, SOT *n* = 6, Other ImSupp *n* = 9) in ICC, SOT and Other ImSupp (calculated as fold change of % of antigen-specific cells over % background of spike-specific cells in the unstimulated control). Red dotted line shows the response threshold. Pie charts show the proportion of ‘responders’ (white = ‘non-responders’). (**E**) Percentage of antigen-specific CD8^+^ T cell of the total CD8^+^ T cell population of ‘responders’ in ICC, SOT and Other ImSupp at <30 (ICC *n* = 4, SOT *n* = 2, Other ImSupp *n* = 3) and >90 days (ICC *n* = 4, SOT *n* = 4, Other ImSupp *n* = 6). (**F**) Percentage of memory-specific subsets of antigen-specific CD8^+^ T cells at <30 (ICC *n* = 4, ImCO *n* = 11) and >90 days (ICC *n* = 4, ImCO *n* = 13) in ICC and ImCo. Statistical significance within paired groups was assessed with the Wilcoxon signed-rank test * *p* < 0.05. Statistical significance amongst different groups was assessed with the Mann–Whitney U test * *p* < 0.05.

Following the analysis of the CD4^+^ T cell compartment, we next focused on the CD8^+^ T cells, using the AIM assay. Antigen-specific CD8^+^ T cells were identified as CD69^+^4-1BB^+^ and their memory subsets were defined based on CCR7 and CD45RA expression (gating strategy in Figure 4A).

All ICC patients mounted a robust CD8^+^ T cell response at both early (<30 days) and late (>90 days) time points (Figure 4B). In contrast, only 5/18 of the ImCo patients responded at <30 days, increasing to 10/15 patients responding at >90 days, with increasing magnitude of antigen-specific response over time (Figure 4B). Among ‘responders’, the percentage of antigen-specific CD8^+^ T cells was relatively similar (Figure 4C). When further subdividing the ImCo group, we observed that the majority of the patients responded in both groups, with relatively similar patterns between the SOT and Other ImSupp in both the magnitude and percentages of antigen-specific CD8^+^ T cells (Figure 4D,E).

Next, we assessed the memory subsets within the antigen-specific CD8^+^ T cells (T_CM_ (CCR7^+^CD45RA^−^), T_EM_ (CCR7^−^CD45RA^−^), T_EMRA_ (CCR7^−^CD45RA^+^), T_SCM-like_ (CCR7^+^CD45RA^+^)) (Figure 4F, gating strategy in Figure 4A). Unlike CD4^+^ T cells, where the T_CM_ subset was predominant, CD8^+^ T_CM_ cells constituted less than 40% of antigen-specific CD8^+^ T cells (Figure 4F). The ICC patients had an increase in T_EM_ frequencies over time, while the ImCo patients had relatively stationary frequencies at the two time points (Figure 4F). CD8^+^ T_EMRA_ cell frequencies decreased over time in ICC patients, whereas the frequencies increased in the ImCo patients at convalescence; however, the variability within the groups is high (Figure 4F). The T_SCM-like_ cell frequencies are relatively similar in the ICC and ImCo patients, with a trend towards lower frequencies in convalescence in the ImCo patients (Figure 4D,E).

These results suggest that even though the majority of the immunosuppressed patients did not mount a SARS-CoV-2-specific CD8^+^ T cell response at the early sampling point, the majority can mount a CD8^+^ T cell response at the later time, demonstrating that immunocompromised patients may also mount an adequate CD8^+^ T cell response.

## 4. Discussion

Immunocompromised patient populations remain at risk of severe COVID-19 due to suboptimal responses to vaccination and natural infection. Despite the partial success of mRNA vaccines in reducing the overall burden of the disease for immunocompromised patients, understanding the immune response in vulnerable patient groups is critical [12,15,18,28]. In this study, we show that the humoral and cellular immune responses may differ depending on immunosuppression, and that antigen-specific T cell responses can still be present despite B cell suppression.

Serological responses are crucial when assessing the ability of a patient to neutralize the virus. The serological responses to SARS-CoV-2 in immunocompetent patients have been well characterized [5,29]. It has been previously reported that increased antibody production but low neutralizing potency of these antibodies correlate with severe COVID-19 in immunocompetent patients [30]. Further, the immune response to breakthrough infections may differ depending on whether patients have hybrid immunity or vaccination-only immunity [31]. Hybrid immunity given by vaccination and natural infection aids in maintaining antibody dependent cellular cytotoxic responses but lower neutralizing capacity [31]. Conversely the response in immunocompromised individuals remains less understood, mainly due to the heterogeneity of immune defects in patients. In a study on immunocompromised patients with SARS-CoV-2 infection, higher levels of neutralizing antibodies decreased viral shedding [32]. In our study, the serological data are complex at the early time points due to confounding factors such as monoclonal antibody distribution and convalescent plasma used to treat the majority of the ImCo patients. However, we found that ImCo patients displayed very low levels of B cells and plasmablasts, as well as IgG and spike-specific mASCs. When SOT patients were subdivided to their own group, we noticed that they present detectable levels of antibody-secreting cells at both early and late time points during the infection. It has been shown in the context of newer variants of concern, that an early higher baseline of SARS-CoV-2-specific B cells and serological response is observed in patients with early viral clearance [33]. Even if we do not have data to support an earlier viral clearance in the SOT patients, we noticed a shorter time of hospitalization for the SOT patients as compared to the other ImCo patients. Further, we observe that SOT patients present significantly higher Spike/ACE2-blocking antibodies at >90 days compared to other immunosuppressed patients. It is important to note that SOT patients in our study had higher vaccination rates compared to the rest of the ImCo group; therefore, it is likely that vaccination influenced the serological response in these patients. Reports have shown that vaccination could induce a robust CD4^+^ and CD8^+^ T cell response, and interestingly it has been reported one vaccination dose provided enhanced T cell responses in case of previous infection [34,35].

The later time point, most likely represents the patients’ intrinsic ability to produce antibodies, as no patient had received any mAb or convalescent plasma for at least 3 months prior to this sampling [26,27]. The detection of N-Ig antibodies in the SOT patients further supports a durable B cell and antibody response in this patient group, similar to a previous study indicating that detectable antibodies can persist for up to at least eight months post-infection in SOT patients [36]. One concern, when treating patients with mAbs during SARS-CoV-2 infection, is that the patient will not be able to mount an endogenous immune response against the virus [37,38]. In an open-labeled clinical trial where the interaction of the mAbs casirivimab and imdevimab with Moderna’s mRNA-1273 was investigated in healthy SARS-CoV-2 individuals, the administration of mAbs before or at the same time as the vaccine lead to a decrease in vaccine-mediated neutralizing antibody levels, whereas total antibodies in serum and cellular immunity were minimally affected [39]. In our cohort, five of the seven SOT patients had received mAbs, indicating that administration of mAbs after infection does not affect the ability of an endogenous immune response in this patient group.

T cell responses in immunocompromised patients, particularly CD8^+^ T cells, have been identified as crucial compensatory mechanisms when humoral immunity is impaired [15,19]. Patients with hematological malignancies have lower CD8^+^ T cell responses compared to those with solid tumors when infected with SARS-CoV-2 [19,40], although robust CD4^+^ T cell responses can still be relevant in viral clearance with a predominant CD4^+^ immunophenotype [21].

In immunocompetent patients, CD4^+^ T cells have been shown to be skewed towards a T_FH_ phenotype, suggesting a critical role in aiding B cells in antibody production [41] that could be impaired in immunosuppression. Immunosuppressed patients showed lower T_FH_ cell proportions as compared to immunocompetent controls, but this difference was not significant.

Immunocompetent patients mounted a robust CD4^+^ and CD8^+^ T cell response, characterized by a predominant T_CM_ profile as previously been reported post-infection [42]. On the other hand, immunocompromised patients were mostly ‘non-responders’ at <30 days, with increasing proportion of ‘responders’ over time. These patterns reflect the complex interplay between condition-specific immunosuppression and the ability to mount a cellular immune response. Studies on hematological malignancies indicate that T cell responses, particularly those of CD8^+^ T cells, remain vital for the survival and recovery of these patients [43]. It is common for patients with hematological malignancies and B cell impaired patients to experience prolonged virus shedding and to display an exhausted CD4^+^ T cell phenotype, leading to a dysfunctional T cell response overall during acute infection [44,45]. In line with our study, patients on anti-CD20 medication have been reported to exhibit robust T cell responses despite their impaired B cell function. Multiple studies demonstrate that following SARS-CoV-2 infection or vaccination, the T cell responses are comparable to those of immunocompetent controls upon peptide stimulation, despite poor B cell responses [15,16,19].

Dissecting the CD4^+^ memory subtypes, we identified that non-responding immunocompromised patients displayed higher proportions of T_SCM-like_ CD4^+^ cells at <30 days. T_SCM-like_ CD4^+^ cells aid in the maintenance of T cell SARS-CoV-2-specific memory and is sustained in immunocompetent patients for 10 months [46]. This finding suggests that T cells may retain some memory despite a lack of immediate response to SARS-CoV-2 infection, warranting further investigation into the role of T_SCM-like_ cells in sustaining T cell memory, particularly in immunocompromised patients.

This study is limited by the heterogeneity of treatments and underlying conditions, as well as a small sample size and the limited biological material. Nevertheless, our findings highlight the critical need to examine immune responses in immunocompromised patients, to better manage this vulnerable group during the emergence of new COVID variants and future viral outbreaks.

Together, our data show that patients with impaired B cell functionality due to immunosuppression can still, to some extent, mount an effective T cell response, particularly through CD8^+^ T cells. This study also highlights that while fewer immunocompromised patients mount a CD4^+^ and CD8^+^ T cell responses at <30 days, these responses tend to increase over time. Importantly, the magnitude of the response is comparable to immunocompetent controls, regardless of immunosuppression. Our study also contributes to the understanding by detailing the differences in T cell subsets in the context of natural SARS-CoV-2 infection, highlighting how patients with an impaired humoral response, still can mount effective T cell responses.

A deeper understanding of the interplay between humoral and cellular immune response in different immunocompromised groups can provide valuable insights into viral clearance and immune responses, not only for SARS-CoV-2 but also for other viral infections.

## Figures and Tables

**Table 1:** Patient characteristics.

	ICC (*n* = 4)	ImCo (*n* = 19)
Age (years), median (range)	65.5 (49–77)	62 (35–78)
Females, *n* (%)	2 (50)	12 (63)
COVID-19 vaccination before SARS-CoV-2 infection, *n* (%)	1 (25)	9 (47)
Vaccination before SARS-CoV-2 infection, number of doses, median (range)	1 (1)	2 (1–3)
Days from vaccination (last dose) until symptom debut, median (range)	1 (1)	19 (1–103)
Patients who received Pfizer-BioNTech™ COVID-19 adenovirus-vectored Vaccine *n* (%)	N/A	9 (47)
Patients who received Vaxzevria™ (Oxford-AstraZeneca) COVID-19 mRNA Vaccine *n* (%)	1 (25)	N/A
Symptom onset to diagnostic PCR sampling, d, median (range)	3 (2–7)	6 (0–17)
Heart transplant *n* (%)	N/A	2 (11)
Kidney transplant *n* (%)	N/A	5 (26)
Multiple sclerosis *n* (%)	N/A	1 (5)
Rheumatoid arthritis *n* (%)	N/A	3 (16)
Kidney failure with nephrosclerosis *n* (%)	N/A	1 (5)
Granulomatosis with polyangiitis *n* (%)	N/A	2 (11)
Myeloma *n* (%)	N/A	2 (11)
Marginal zone lymphoma *n* (%)	N/A	1 (5)
Myelodysplastic syndrome with multilineage dysplasia *n* (%)	N/A	1 (5)
Chronic lymphocytic leukemia *n* (%)	N/A	2 (11)
Rituximab, *n* (%)	N/A	7 (37)
Days since last RTX dose at symptom debut, median (range)	N/A	69 (4–134)
TNF-α inhibitor *n* (%)	N/A	1 (5)
Daratumumab *n* (%)	N/A	2 (11)
Tacrolimus *n* (%)	N/A	7 (37)
Everolimus *n* (%)	N/A	1 (5)
Steroids *n* (%)	N/A	9 (11)
Steroids (mg/day) median (range)	N/A	5 (3–10)
Mycophenol or methotrexate *n* (%)	N/A	8 (42)
Small molecules *n* (%)	N/A	3 (16)
Other immunosuppressants *n* (%)	N/A	1 (5)
Years since hemopathy diagnosis at symptom debut, median (range)	N/A	7 (5–25)
Years since solid organ transplantation at symptom debut	N/A	5 (<1–16)
Days since last RTX dose at symptom debut, median (range)	N/A	69 (4–134)

**Table 2:** Clinical information.

	ICC (*n* = 4)	ImCo (*n* = 19)
Day of sampling <30 days after symptom debut Median (range)	19 (15–27)	15 (4–22)
Day of sampling >90 days after symptom debut Median (range)	169 (158–176)	182 (99–265)
Days of hospitalization Median (range)	13 (10–22)	10 (1–38)
Patients in ICU *n* (%)	2 (50)	2 (11)
Mild disease with need of assistance *n* (%)	N/A	3 (16)
Moderate disease; no oxygen therapy *n* (%)	N/A	10 (53)
Moderate disease; oxygen by mask *n* (%)	3 (75)	6 (32)
Severe disease; oxygen by non-invasive ventilation *n* (%)	1 (25)	N/A
Diabetes mellitus *n* (%)	2 (50)	7 (37)
Hypertonia *n* (%)	4 (100)	14 (74)
Heart failure *n* (%)	N/A	3 (16)
Lung disease (asthma, chronic bronchitis, chronic obstructive pulmonary disease) *n* (%)	N/A	8 (42)
Kidney insufficiency *n* (%)	N/A	10 (53)
Other *	1 (25)	7 (37)
BMI >30 *n* (%)	2 (50)	5 (26)
Smoker *n* (%)	N/A	4 (21)
Positive virus culture from patient airway material *n* (%)	2 (50)	8 (42)
of positive samples in culture, positive for CPE *n* (%)	0	2 (11)
Monoclonal antibody (mAb) therapy *n* (%)	1 (25)	8 (42)
mAb therapy received how many days before <30 day sampling median (range)	8 (8)	3 (0–8)
mAb therapy received how many days before >90 day sampling median (range)	167 (167)	162 (89–177)
Convalescent plasma (cPlasma) treatment *n* (%)	0	8 (42)
cPlasma therapy received how many days before <30 day sampling median (range)	N/A	4 (0–10)
cPlasma therapy received how many days before >90 day sampling median (range)	N/A	180 (126–229)
Remdesivir treatment *n* (%)	1 (25)	11 (58)
Remdesivir received how many days before <30 day sampling median (range)	6 (6)	6 (3–24)
Remdesivir received how many days before >90 day sampling median (range)	168 (168)	181 (126–245)
Corticosteroids treatment *n* (%)	4 (100)	13 (68)
Corticosteroids received how many days before <30 day sampling median (range)	9 (8–17)	12 (4–37)
Corticosteroids received how many days before >90 day sampling median (range)	160 (145–168)	180 (89–245)

* Monoclonal gammopathy of undetermined significance, Necrobiotic Xanthogranuloma, previous breast cancer, hypothyroidism, and hypogammaglobulinemia.

## Data Availability

The data that support the findings of this study are available on request from the corresponding author. The data are not publicly available because of privacy or ethical restrictions.

## Supplementary Materials

The following supporting information can be downloaded at: https://www.mdpi.com/article/10.3390/microorganisms13051077/s1, Graphical abstract was created in BioRender Mayola, N. (2025) https://BioRender.com/r22x907 (accessed on 13 March 2025). Figure S1: (A) Identification of immune cell subsets in immunocompetent and immunocompromised COVID-19 patients. Gating strategy to identify major immune cell subsets in peripheral blood for analysis of TruCount data. Gated cell subsets are indicated with black numbers, cell frequencies with blue numbers. (B) Monocytes subtypes absolute numbers per μL of whole blood at <30 (ICC *n* = 4, ImCo *n* = 17) and >90 days (ICC *n* = 4, ImCo *n* = 15). cMono: classical monocytes, iMono: intermediate monocytes, ncMono: non-classical monocytes. Plots depict median with IQR. (C) Dendritic cell subtypes’ numbers, per μL of whole blood in ICC and ImCo. cDC: conventional dendritic cells, pDC: plasmacytoid dendritic cells at <30 (ICC *n* = 4, ImCo *n* = 17) and >90 days (ICC *n* = 4, ImCo *n* = 15). Plots depict median with IQR. Statistical significance within paired groups was assessed with Wilcoxon signed-rank test * *p* < 0.05. Statistical significance amongst different groups was assessed with Mann–Whitney U test. Figure S2: Treatment and hospitalization in immunosuppressed COVID-19 patients (A) Pie charts of the proportion of COVID-19 patients that received monoclonal antibody treatment, convalescent plasma treatment, Remdesivir, or corticosteroid to treat the infection at <30 days. (B) Pie charts of the proportion of COVID-19 patients that received monoclonal antibody treatment, convalescent plasma treatment, Remdesivir, or corticosteroid to treat the infection at >90 days. (C) Bar plots depicting median and IQR CT values from SARS-Cov-2 RNA PCR from airway samples divided into groups of in vitro negative (*n* = 11) and positive (*n* = 10) culture of the airway samples (all at <30 days)**.** Bar plot depicting median and IQR CT values for SARS-CoV-2 RNA PCR from airway samples divided into groups of in vitro visual CPE negative (*n* = 2) and CPE positive (*n* = 8) (all at <30 days). (D) Heatmap showing the SARS-CoV-2 variants in the patients. Figure S3: Antibody responses in patients divided into groups based on treatment. (A) Spike/ACE2 blocking antibodies at <30 days (ICC (*n* = 3) vs. Non-mAb ImCo (*n* = 9) and mAb ImCo (*n* = 8) and >90 days (ICC (*n* = 3) vs. Non-mAb ImCo (*n* = 7) and mAb ImCo (*n* = 5). The dotted lines denote the positive threshold (≥0.533 μg/mL) and upper detection limit (12 μg/mL). (B) Spike IgG levels at <30 days (ICC (*n* = 3) vs. Non-mAb ImCo (*n* = 10) and mAb ImCo (*n* = 8) and >90 days (ICC (*n* = 3) vs. Non-mAb ImCo (*n* = 7) and mAb ImCo (*n* = 8). The dotted line denotes the positive threshold (17.7 BAU/mL). (C) Nucleocapsid specific IgG <30 days (ICC (*n* = 3) vs. Non-mAb ImCo (*n* = 10) and mAb ImCo (*n* = 8) and >90 days (ICC (*n* = 3) vs. Non-mAb ImCo (*n* = 7) and mAb ImCo (*n* = 8). The dotted line denotes the positive threshold (11.8 BAU/mL). ICC patient that received mAb has been excluded from analysis. (D) Spike/ACE2 blocking antibodies at <30 days (ICC (*n* = 4) vs. non-convalescent plasma (cPlasma ImCo (*n* = 10) and cPlasma ImCo (*n* = 6) and >90 days (ICC (*n* = 4) vs. Non-cPlasma ImCo (*n* = 7) and cPlasma ImCo (*n* = 6). The dotted lines denote the positive threshold (≥0.533 μg/mL) and upper detection limit (12 μg/mL). (E) Spike IgG levels at <30 days (ICC (*n* = 4) vs. Non-cPlasma ImCo (*n* = 13) and cPlasma ImCo (*n* = 6) and >90 days (ICC (*n* = 4) vs. Non-cPlasma ImCo (*n* = 10) and cPlasma ImCo (*n* = 6). The dotted line denotes the positive threshold (17.7 BAU/mL). (F) Nucleocapsid specific IgG at <30 days (ICC (*n* = 4) vs. Non-cPlasma ImCo (*n* = 12) and cPlasma ImCo (*n* = 6) and >90 days (ICC (*n* = 4) vs. Non-cPlasma ImCo (*n* = 9) and cPlasma ImCo (*n* = 6). The dotted line denotes the positive threshold (11.8 BAU/mL). Statistical significance within paired groups was assessed with Wilcoxon signed-rank test * *p* < 0.05. Statistical significance amongst different groups was assessed with Mann-Whitney U test * *p* < 0.05. Table S1: primers.

## References

[1] Evans R.A., Dube S., Lu Y., Yates M., Arnetorp S., Barnes E., Bell S., Carty L., Evans K., Graham S. (2023). Impact of COVID-19 on Immunocompromised Populations during the Omicron Era: Insights from the Observational Population-Based INFORM Study. Lancet Reg. Health Eur..

[2] Bertini C.D., Khawaja F., Sheshadri A. (2023). Coronavirus Disease-2019 in the Immunocompromised Host. Clin. Chest Med..

[3] Pinato D.J., Aguilar-Company J., Ferrante D., Hanbury G., Bower M., Salazar R., Mirallas O., Sureda A., Plaja A., Cucurull M. (2022). Outcomes of the SARS-CoV-2 Omicron (B.1.1.529) Variant Outbreak among Vaccinated and Unvaccinated Patients with Cancer in Europe: Results from the Retrospective, Multicentre, OnCovid Registry Study. Lancet Oncol..

[4] Chen X., Pan Z., Yue S., Yu F., Zhang J., Yang Y., Li R., Liu B., Yang X., Gao L. (2020). Disease Severity Dictates SARS-CoV-2-Specific Neutralizing Antibody Responses in COVID-19. Signal Transduct. Target. Ther..

[5] Marcotte H., Piralla A., Zuo F., Du L., Cassaniti I., Wan H., Kumagai-Braesh M., Andréll J., Percivalle E., Sammartino J.C. (2022). Immunity to SARS-CoV-2 up to 15 Months after Infection. iScience.

[6] Pitiriga V.C., Papamentzelopoulou M., Konstantinakou K.E., Vasileiou I.V., Sakellariou K.S., Spyrou N.I., Tsakris A. (2023). Persistence of T-Cell Immunity Responses against SARS-CoV-2 for over 12 Months Post COVID-19 Infection in Unvaccinated Individuals with No Detectable IgG Antibodies. Vaccines.

[7] Zuo J., Dowell A.C., Pearce H., Verma K., Long H.M., Begum J., Aiano F., Amin-Chowdhury Z., Hoschler K., Brooks T. (2021). Robust SARS-CoV-2-Specific T Cell Immunity Is Maintained at 6 Months Following Primary Infection. Nat. Immunol..

[8] Dan J.M., Mateus J., Kato Y., Hastie K.M., Yu E.D., Faliti C.E., Grifoni A., Ramirez S.I., Haupt S., Frazier A. (2021). Immunological Memory to SARS-CoV-2 Assessed for up to 8 Months after Infection. Science.

[9] Rahmani K., Shavaleh R., Forouhi M., Disfani H.F., Kamandi M., Oskooi R.K., Foogerdi M., Soltani M., Rahchamani M., Mohaddespour M. (2022). The Effectiveness of COVID-19 Vaccines in Reducing the Incidence, Hospitalization, and Mortality from COVID-19: A Systematic Review and Meta-Analysis. Front. Public. Health.

[10] Bergman P., Blennow O., Hansson L., Mielke S., Nowak P., Chen P., Söderdahl G., Österborg A., Smith C.I.E., Wullimann D. (2021). Safety and Efficacy of the mRNA BNT162b2 Vaccine against SARS-CoV-2 in Five Groups of Immunocompromised Patients and Healthy Controls in a Prospective Open-Label Clinical Trial. EBioMedicine.

[11] Dib M., Le Corre N., Ortiz C., García D., Ferrés M., Martinez-Valdebenito C., Ruiz-Tagle C., Ojeda M.J., Espinoza M.A., Jara A. (2022). SARS-CoV-2 Vaccine Booster in Solid Organ Transplant Recipients Previously Immunised with Inactivated versus mRNA Vaccines: A Prospective Cohort Study. Lancet Reg. Health Am..

[12] Sakuraba A., Luna A., Micic D. (2022). A Systematic Review and Meta-Analysis of Serologic Response Following Coronavirus Disease 2019 (COVID-19) Vaccination in Solid Organ Transplant Recipients. Viruses.

[13] Napuri N.I., Curcio D., Swerdlow D.L., Srivastava A. (2022). Immune Response to COVID-19 and mRNA Vaccination in Immunocompromised Individuals: A Narrative Review. Infect. Dis. Ther..

[14] Bytyci J., Ying Y., Lee L.Y.W. (2024). Immunocompromised Individuals Are at Increased Risk of COVID-19 Breakthrough Infection, Hospitalization, and Death in the Post-Vaccination Era: A Systematic Review. Immun. Inflamm. Dis..

[15] Apostolidis S.A., Kakara M., Painter M.M., Goel R.R., Mathew D., Lenzi K., Rezk A., Patterson K.R., Espinoza D.A., Kadri J.C. (2021). Cellular and Humoral Immune Responses Following SARS-CoV-2 mRNA Vaccination in Patients with Multiple Sclerosis on Anti-CD20 Therapy. Nat. Med..

[16] Madelon N., Lauper K., Breville G., Sabater Royo I., Goldstein R., Andrey D.O., Grifoni A., Sette A., Kaiser L., Siegrist C.A. (2022). Robust T-Cell Responses in Anti-CD20-Treated Patients Following COVID-19 Vaccination: A Prospective Cohort Study. Clin. Infect. Dis..

[17] Mrak D., Tobudic S., Koblischke M., Graninger M., Radner H., Sieghart D., Hofer P., Perkmann T., Haslacher H., Thalhammer R. (2021). SARS-CoV-2 Vaccination in Rituximab-Treated Patients: B Cells Promote Humoral Immune Responses in the Presence of T-Cell-Mediated Immunity. Ann. Rheum. Dis..

[18] Gao Y., Cai C., Wullimann D., Niessl J., Rivera-Ballesteros O., Chen P., Lange J., Cuapio A., Blennow O., Hansson L. (2022). Immunodeficiency Syndromes Differentially Impact the Functional Profile of SARS-CoV-2-Specific T Cells Elicited by mRNA Vaccination. Immunity.

[19] Bange E.M., Han N.A., Wileyto P., Kim J.Y., Gouma S., Robinson J., Greenplate A.R., Hwee M.A., Porterfield F., Owoyemi O. (2021). CD8+ T Cells Contribute to Survival in Patients with COVID-19 and Hematologic Cancer. Nat. Med..

[20] Barnes E., Goodyear C.S., Willicombe M., Gaskell C., Siebert S., I de Silva T., Murray S.M., Rea D., Snowden J.A., Carroll M. (2023). SARS-CoV-2-Specific Immune Responses and Clinical Outcomes after COVID-19 Vaccination in Patients with Immune-Suppressive Disease. Nat. Med..

[21] Lyudovyk O., Kim J.Y., Qualls D., Hwee M.A., Lin Y.-H., Boutemine S.R., Elhanati Y., Solovyov A., Douglas M., Chen E. (2022). Impaired Humoral Immunity Is Associated with Prolonged COVID-19 despite Robust CD8 T Cell Responses. Cancer Cell.

[22] (2023). Intensivvårdade Fall av COVID-19 Under Tidsperioder Med Olika Dominerande Virusvarianter [Intensive Care Cases of COVID-19 During Periods with Different Dominant Virus Variants].

[23] Marshall J.C., Murthy S., Diaz J., Adhikari N.K., Angus D.C., Arabi Y.M., Baillie K., Bauer M., Berry S., Blackwood B. (2020). A Minimal Common Outcome Measure Set for COVID-19 Clinical Research. Lancet Infect. Dis..

[24] Corman V.M., Landt O., Kaiser M., Molenkamp R., Meijer A., Chu D.K., Bleicker T., Brünink S., Schneider J., Schmidt M.L. (2020). Detection of 2019 Novel Coronavirus (2019-nCoV) by Real-Time RT-PCR. Euro Surveill..

[25] Sandberg J.T., Varnaitė R., Christ W., Chen P., Muvva J.R., Maleki K.T., García M., Dzidic M., Folkesson E., Skagerberg M. (2021). SARS-CoV-2-Specific Humoral and Cellular Immunity Persists through 9 Months Irrespective of COVID-19 Severity at Hospitalisation. Clin. Transl. Immunol..

[26] Stadler E., Burgess M.T., Schlub T.E., Khan S.R., Chai K.L., McQuilten Z.K., Wood E.M., Polizzotto M.N., Kent S.J., Cromer D. (2023). Monoclonal Antibody Levels and Protection from COVID-19. Nat. Commun..

[27] Huygens S., Preijers T., Swaneveld F.H., Kleine Budde I., GeurtsvanKessel C.H., Koch B.C.P., Rijnders B.J.A. (2024). Dosing of Convalescent Plasma and Hyperimmune Anti-SARS-CoV-2 Immunoglobulins: A Phase I/II Dose-Finding Study. Clin. Pharmacokinet..

[28] Plasencia-Rodríguez C., Martínez-Feito A., Hernández M., Del Pino-Molina L., Novella-Navarro M., Serrano Y., González-Muñoz M., Peiteado D., Bonilla G., Monjo I. (2023). Immune Response after SARS-CoV-2 Vaccination in Patients with Inflammatory Immune-Mediated Diseases Receiving Immunosuppressive Treatment. Allergy Asthma Clin. Immunol..

[29] Scharf L., Axelsson H., Emmanouilidi A., Mathew N.R., Sheward D.J., Leach S., Isakson P., Smirnov I.V., Marklund E., Miron N. (2023). Longitudinal Single-Cell Analysis of SARS-CoV-2–Reactive B Cells Uncovers Persistence of Early-Formed, Antigen-Specific Clones. JCI Insight.

[30] Garcia-Beltran W.F., Lam E.C., Astudillo M.G., Yang D., Miller T.E., Feldman J., Hauser B.M., Caradonna T.M., Clayton K.L., Nitido A.D. (2021). COVID-19-Neutralizing Antibodies Predict Disease Severity and Survival. Cell.

[31] Saade C., Bruel T., Vrignaud L.-L., Killian M., Drouillard A., Barateau V., Espi M., Mariano N., Mignon C., Bruyère L. (2025). BA.1 Breakthrough Infection Elicits Distinct Antibody and Memory B Cell Responses in Vaccinated-Only versus Hybrid Immunity Individuals. iScience.

[32] Lim S.Y., Kim J.-W., Kim J.Y., Kang S.-W., Jang C.-Y., Chang E., Yang J.-S., Kim K.-C., Jang H.-C., Kim D.S. (2024). The Association Between Antibody Responses and Prolonged Viable Severe Acute Respiratory Syndrome Coronavirus 2 Shedding in Immunocompromised Patients: A Prospective Cohort Study. J. Infect. Dis..

[33] Vergouwe M., Biemond J.J., van der Straten K., van Pul L., Kerster G., Claireaux M., Burger J.A., van Dort K.A., Kootstra N.A., Jonges M. (2024). A Robust Severe Acute Respiratory Syndrome Coronavirus 2 (SARS-CoV-2)–Specific T- and B-Cell Response Is Associated With Early Viral Clearance in SARS-CoV-2 Omicron-Infected Immunocompromised Individuals. J. Infect. Dis..

[34] Mazzoni A., Di Lauria N., Maggi L., Salvati L., Vanni A., Capone M., Lamacchia G., Mantengoli E., Spinicci M., Zammarchi L. (2021). First-Dose mRNA Vaccination Is Sufficient to Reactivate Immunological Memory to SARS-CoV-2 in Subjects Who Have Recovered from COVID-19. J. Clin. Investig..

[35] Sahin U., Muik A., Vogler I., Derhovanessian E., Kranz L.M., Vormehr M., Quandt J., Bidmon N., Ulges A., Baum A. (2021). BNT162b2 Vaccine Induces Neutralizing Antibodies and Poly-Specific T Cells in Humans. Nature.

[36] Favà A., Donadeu L., Jouve T., Gonzalez-Costello J., Lladó L., Santana C., Toapanta N., Lopez M., Pernin V., Facundo C. (2022). A Comprehensive Assessment of Long-Term SARS-CoV-2–Specific Adaptive Immune Memory in Convalescent COVID-19 Solid Organ Transplant Recipients. Kidney Int..

[37] Kim P.S., Dimcheff D.E., Siler A., Schildhouse R.J., Chensue S.W. (2022). Effect of Monoclonal Antibody Therapy on the Endogenous SARS-CoV-2 Antibody Response. Clin. Immunol..

[38] Zhang L., Poorbaugh J., Dougan M., Chen P., Gottlieb R.L., Huhn G., Beasley S., Daniels M., Ngoc Vy Trinh T., Crisp M. (2021). Endogenous Antibody Responses to SARS-CoV-2 in Patients With Mild or Moderate COVID-19 Who Received Bamlanivimab Alone or Bamlanivimab and Etesevimab Together. Front. Immunol..

[39] Isa F., Gonzalez Ortiz A.M., Meyer J., Hamilton J.D., Olenchock B.A., Brackin T., Ganguly S., Forleo-Neto E., Faria L., Heirman I. (2025). Effect of Timing of Casirivimab and Imdevimab Administration Relative to mRNA-1273 COVID-19 Vaccination on Vaccine-Induced SARS-CoV-2 Neutralising Antibody Responses: A Prospective, Open-Label, Phase 2, Randomised Controlled Trial. Lancet Infect. Dis..

[40] Fendler A., Au L., Shepherd S.T.C., Byrne F., Cerrone M., Boos L.A., Rzeniewicz K., Gordon W., Shum B., Gerard C.L. (2021). Functional Antibody and T Cell Immunity Following SARS-CoV-2 Infection, Including by Variants of Concern, in Patients with Cancer: The CAPTURE Study. Nat. Cancer.

[41] Sekine T., Perez-Potti A., Rivera-Ballesteros O., Strålin K., Gorin J.-B., Olsson A., Llewellyn-Lacey S., Kamal H., Bogdanovic G., Muschiol S. (2020). Robust T Cell Immunity in Convalescent Individuals with Asymptomatic or Mild COVID-19. Cell.

[42] Gao Y., Cai C., Grifoni A., Müller T.R., Niessl J., Olofsson A., Humbert M., Hansson L., Österborg A., Bergman P. (2022). Ancestral SARS-CoV-2-Specific T Cells Cross-Recognize the Omicron Variant. Nat. Med..

[43] Ingelman-Sundberg H.M., Blixt L., Wullimann D., Wu J., Gao Y., Healy K., Muschiol S., Bogdanovic G., Åberg M., Kjellander C. (2023). Systemic and Mucosal Adaptive Immunity to SARS-CoV-2 during the Omicron Wave in Patients with Chronic Lymphocytic Leukemia. Haematologica.

[44] Abdul-Jawad S., Baù L., Alaguthurai T., del Molino del Barrio I., Laing A.G., Hayday T.S., Monin L., Muñoz-Ruiz M., McDonald L., Francos Quijorna I. (2021). Acute Immune Signatures and Their Legacies in Severe Acute Respiratory Syndrome Coronavirus-2 Infected Cancer Patients. Cancer Cell.

[45] Raglow Z., Surie D., Chappell J.D., Zhu Y., Martin E.T., Kwon J.H., Frosch A.E., Mohamed A., Gilbert J., Bendall E.E. (2024). SARS-CoV-2 Shedding and Evolution in Patients Who Were Immunocompromised during the Omicron Period: A Multicentre, Prospective Analysis. Lancet Microbe.

[46] Jung J.H., Rha M.-S., Sa M., Choi H.K., Jeon J.H., Seok H., Park D.W., Park S.-H., Jeong H.W., Choi W.S. (2021). SARS-CoV-2-Specific T Cell Memory Is Sustained in COVID-19 Convalescent Patients for 10 Months with Successful Development of Stem Cell-like Memory T Cells. Nat. Commun..

