# Supplementary material for: Dysregulated Adaptive Immune Responses to SARS-CoV-2 in Immunocompromised Individuals
Source: Microorganisms. 2025 May 6;13(5):1077. doi: 10.3390/microorganisms13051077 (PMC12114339; doi:10.3390/microorganisms13051077)
Supplement: Supplementary file 1 [file microorganisms-13-01077-s001.zip › microorganisms-3558074-supplementary.pdf]

## Supplementary figure legends

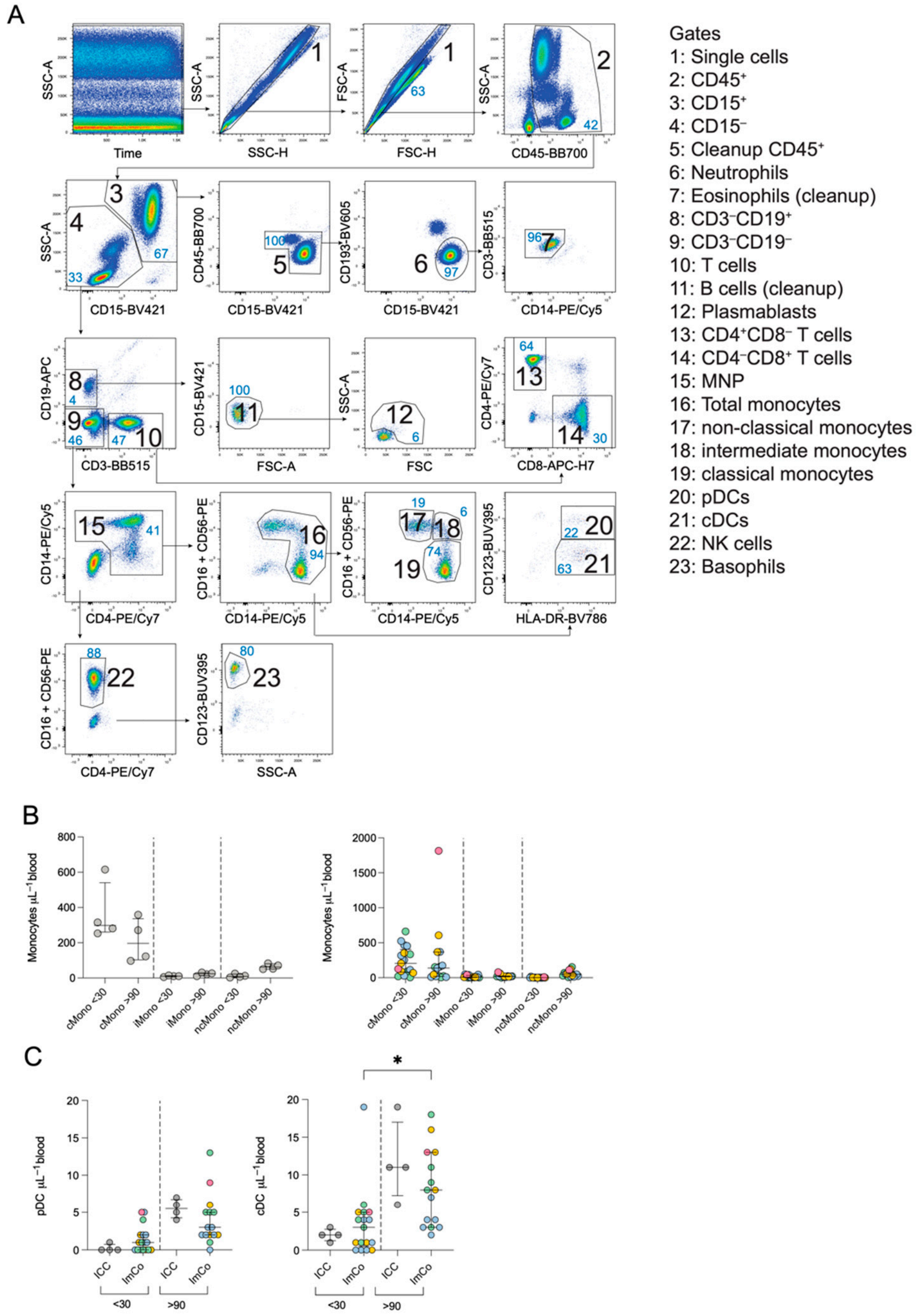


Figure S1: **A)** Identification of immune cell subsets in immunocompetent and immunocompromised COVID-19 patients. Gating strategy to identify major immune cell

subsets in peripheral blood for analysis of TruCount data. Gated cell subsets are indicated with black numbers, cell frequencies with blue numbers. **B)** Monocytes subtypes absolute numbers per µL of whole blood at <30 (ICC n=4, ImCo n=17) and >90 days (ICC n=4, ImCo n=15). cMono: classical monocytes, iMono: intermediate monocytes, ncMono: non-classical monocytes. Plots depict median with IQR. **C)** Dendritic cell subtypes' numbers, per µL of whole blood in ICC and ImCo. cDC: conventional dendritic cells, pDC: plasmacytoid dendritic cells at <30 (ICC n=4, ImCo n=17) and >90 days (ICC n=4, ImCo n=15). Plots depict median with IQR.

Statistical significance within paired groups was assessed with Wilcoxon signed-rank test *$p<0.05$. Statistical significance amongst different groups was assessed with Mann–Whitney U test.

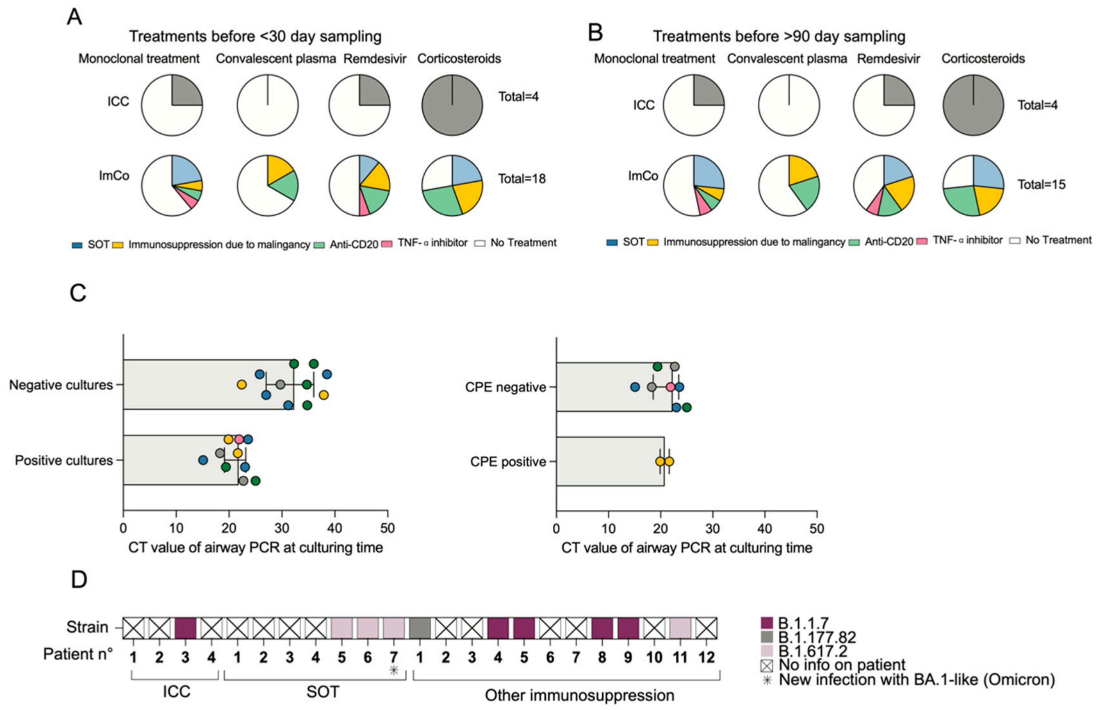


Figure S2**:** Treatment and hospitalization in immunosuppressed COVID-19 patients **A)** Pie charts of the proportion of COVID-19 patients that received monoclonal antibody treatment, convalescent plasma treatment, Remdesivir, or corticosteroid to treat the infection at <30 days

**B)** Pie charts of the proportion of COVID-19 patients that received monoclonal antibody treatment, convalescent plasma treatment, Remdesivir, or corticosteroid to treat the infection at >90 days. **C)** Bar plots depicting median and IQR CT values from SARS-Cov-2 RNA PCR from airway samples divided into groups of *in vitro* negative (n=11) and positive (n=10) culture of the airway samples (all at <30 days). Bar plot depicting median and IQR CT values for SARS-CoV-2 RNA PCR from airway samples divided into groups of *in vitro* visual CPE negative (n=2) and CPE positive (n=8) (all at <30 days). **D)** Heatmap showing the SARS-CoV-2 variants in the patients.

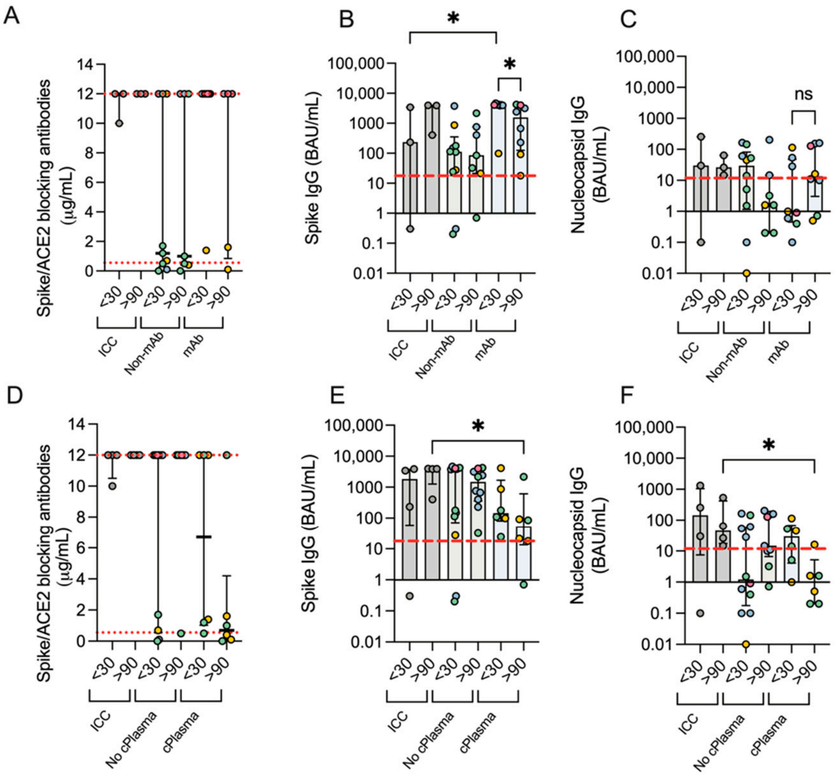


Figure S3**:** Antibody responses in patients divided into groups based on treatment**. A)** Spike/ACE2 blocking antibodies at <30 days (ICC (n=3) vs Non-mAb ImCo (n=9) and mAb ImCo (n=8) and >90 days (ICC (n=3) vs Non-mAb ImCo (n=7) and mAb ImCo (n=5). The

dotted lines denote the positive threshold (≥0.533 µg/mL) and upper detection limit (12µg/mL). **B)** Spike IgG levels at <30 days (ICC (n=3) vs Non-mAb ImCo (n=10) and mAb ImCo (n=8) and >90 days (ICC (n=3) vs Non-mAb ImCo (n=7) and mAb ImCo (n=8). The dotted line denotes the positive threshold (17.7 BAU/mL). **C)** Nucleocapsid specific IgG <30 days (ICC (n=3) vs Non-mAb ImCo (n=10) and mAb ImCo (n=8) and >90 days (ICC (n=3) vs Non-mAb ImCo (n=7) and mAb ImCo (n=8). The dotted line denotes the positive threshold (11.8 BAU/mL). ICC patient that received mAb has been excluded from analysis **D)** Spike/ACE2 blocking antibodies at <30 days (ICC (n=4) vs non-convalescent plasma (cPlasma ImCo (n=10) and cPlasma ImCo (n=6) and >90 days (ICC (n=4) vs Non-cPlasma ImCo (n=7) and cPlasma ImCo (n=6). The dotted lines denote the positive threshold (≥0.533 µg/mL) and upper detection limit (12µg/mL). **E)** Spike IgG levels at <30 days (ICC (n=4) vs Non-cPlasma ImCo (n=13) and cPlasma ImCo (n=6) and >90 days (ICC (n=4) vs Non-cPlasma ImCo (n=10) and cPlasma ImCo (n=6). The dotted line denotes the positive threshold (17.7 BAU/mL). **F)** Nucleocapsid specific IgG at <30 days (ICC (n=4) vs Non-cPlasma ImCo (n=12) and cPlasma ImCo (n=6) and >90 days (ICC (n=4) vs Non-cPlasma ImCo (n=9) and cPlasma ImCo (n=6). The dotted line denotes the positive threshold (11.8 BAU/mL).

Statistical significance within paired groups was assessed with Wilcoxon signed-rank test *$p$<0.05. Statistical significance amongst different groups was assessed with Mann-Whitney U test *$p$< 0.05.

Table S1: primers

| | |
|---|---|
| **RdRP_SARSr_F3** | **CATGTGTGGCGGTTCACTATATGT** |
| **RdRP_SARSr_R5** | TGTTAAARACACTATTAGCATAWGCAGT |
| **RdRP_SARSr_P2** | CAGGTGGAACCTCATCAGGAGATGC |
